# Systematic review with meta-analysis: age-related malignancy detection rates at upper gastrointestinal endoscopy

**DOI:** 10.1177/1756284820959225

**Published:** 2020-11-04

**Authors:** Judith J. de Jong, Marten A. Lantinga, Ina M. E. Thijs, Philip R. de Reuver, Joost P. H. Drenth

**Affiliations:** Department of Gastroenterology and Hepatology, Radboud University Medical Centre, Nijmegen, The Netherlands; Department of Gastroenterology and Hepatology, Radboud University Medical Centre, Nijmegen, The Netherlands; Department of Gastroenterology and Hepatology, Radboud University Medical Centre, Nijmegen, The Netherlands; Department of Surgery, Radboud University Medical Centre, Nijmegen, The Netherlands; Department of Gastroenterology and Hepatology, Radboud University Medical Center, P.O. Box 9101, Nijmegen, 6500 HB, The Netherlands

**Keywords:** epidemiology, healthcare evaluation, upper gastrointestinal malignancy

## Abstract

**Background:**

Age is an important and objective risk factor for upper gastrointestinal (GI) malignancy. The accuracy of various age limits to detect upper GI malignancy is unclear. Determination of this accuracy may aid in the decision to refer symptomatic patients for upper GI endoscopy. The aim of this analysis was to synthesize data on upper GI malignancy detection rates for various age limits worldwide through meta-analysis.

**Methods:**

We searched MEDLINE, EMBASE, and Web of Science in November 2018. Selection criteria included studies addressing malignant findings at upper GI endoscopy in a symptomatic population reporting age at time of diagnosis. Meta-analyses were conducted to derive continent-specific cancer detection rates.

**Results:**

A total of 33 studies including 346,641 patients across 21 countries fulfilled the inclusion criteria. To detect >80% of malignant cases all symptomatic patients over 40 years of age should be investigated in Africa, over 50 years of age in South America and Asia, and over 55 years of age in North America and Europe.

**Conclusion:**

This systematic review and meta-analysis provides data on intercontinental variation in age at time of upper GI malignancy diagnosis in symptomatic patients referred for upper GI endoscopy. Guideline recommendations for age-based selection should be tailored to local age-related detection rates.

## Introduction

Upper gastrointestinal (GI) tract malignancy is an important cause of mortality, although regional differences exist. In 2018, over 50,000 Europeans were diagnosed with esophageal cancer.^1^ In Asia an eight-fold number of cases was diagnosed. Gastric cancer was diagnosed in over a million patients worldwide in 2018.

Upper GI endoscopy is the universal gold standard for detecting upper GI malignancy.^2,3^ However, the lack of malignancy-specific symptoms results in diagnostic overuse as more than 100 procedures are needed to detect a single patient with a GI tumor.^4^ On the other hand, malignancies may be missed at upper GI endoscopy.^5^ Most procedures are performed for dyspepsia, for which clinically significant findings are detected in <25%.^6^ Several ‘red flag’ symptoms are frequently used to differentiate between those at higher or lower risk of malignancy. However, these symptoms are often nonspecific and subject to interpretation. Age limits, in particular minimum age thresholds, are used in many guidelines to optimize diagnostic yield of upper GI endoscopy. An illustrative example is the guideline of the joint American College of Gastroenterology and Canadian Association of Gastroenterology on dyspepsia management.^7^ This guideline sets an age limit of 60 years and does not advocate upper GI endoscopy as common practice in younger patients.

The impact of age limits on upper GI malignancy detection rate is poorly investigated. Therefore, we performed a systematic review and meta-analysis of studies reporting age at upper GI malignancy diagnosis in a symptomatic population that underwent upper GI endoscopy. Our aim was to calculate the implications of various age limits on region-specific malignancy detection rates.

## Materials and methods

For this systematic review and meta-analysis, we adopted the Preferred Reporting Items for Systematic Reviews and Meta-Analysis 2009 guideline (Supplemental Table 1).^8^ The study protocol is registered in PROSPERO, (CRD42018100060, https://www.crd.york.ac.uk/prospero/). Study selection, data extraction, and quality assessment were independently performed by JJJ and IMET and discrepancies resolved through discussion with MAL. This study was exempted from Institutional Review Board (IRB) approval.

### Search strategy and study selection

The electronic databases MEDLINE (US National Library of Medicine, 1946–2018), EMBASE (Elsevier, 1974–2018), and Web of Science (Clarivate analytics, 1900–2018) were searched to select eligible studies in November 2018 by JJdeJ and IMET. Terms for ‘upper gastrointestinal endoscopy’, ‘upper gastrointestinal malignancy’, and ‘upper gastrointestinal symptoms’ were combined with set operator ‘AND’ (Supplemental Table 2). No limits were applied. Reference lists were scanned for additional studies.

**Figure 1.**
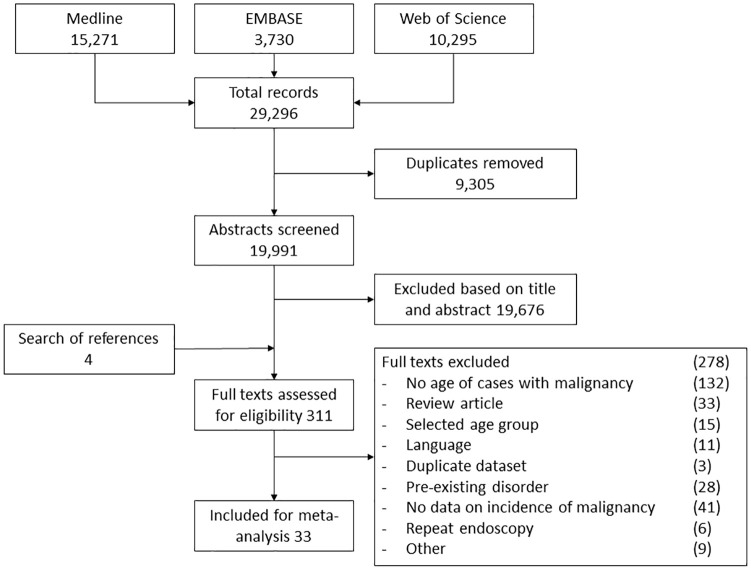
Flow diagram of study selection.

Studies reporting findings of upper GI endoscopy in adults (⩾18 years) were selected for full-text review. Study selection and inclusion and exclusion criteria are presented in Figure 1. We limited our selection to studies published in the year 2000 or thereafter to improve representativeness of results. We defined ‘symptomatic’ as any symptom with a potential origin in the upper GI tract, as opposed to a screening (i.e. asymptomatic) population. Alarm symptoms comprised one the following symptoms; (a) dysphagia; (b) signs of potential upper GI blood loss (hematemesis, melena, anemia, hematochezia); (c) unintentional weight loss; (d) persistent vomiting. We considered conference abstracts for inclusion if data for primary and secondary outcomes were available. If identical populations were reported in multiple studies, we included the most recent study.

### Data extraction

Data were recorded in prespecified data extraction forms. We extracted data on authorship, country, period, design of study, sample size, percentage male, type of symptoms, number of patients with malignancy (gastric *versus* esophageal), and patient’s age at diagnosis of malignancy. If numbers of malignancies were presented in bar charts without textual presentation, we estimated numbers with highest possible precision.

### Data synthesis and statistical analysis

We calculated the prevalence of malignancy with age limits set at 40, 45, 50, 55, 60, and 65 years for each world continent. Prevalence odds ratios (PORs) were calculated using log transformations of the OR of prevalence of malignancy above and below age limits. Of all cases diagnosed with malignancy, we calculated the proportion of cases detected above and below each age limit. A sensitivity of 80% was used as the threshold in presentation of the results to facilitate test accuracy interpretation, in accordance with sensitivity of well-established screening tests such as the fecal immunochemical testing for colorectal neoplasia.^9^ Numbers needed to endoscope above and below age limits to detect one case of malignancy were calculated for all continents. This reflected the proportion of patients above the age limit with malignancy, and the proportion of those below the age limit. Freeman–Tukey double arcsine transformations were applied to all proportions before pooling for stabilization of variance and to allow the inclusion of studies with 0% or 100% prevalence.^10^ Transformed proportions were pooled using a random-effects model, because of heterogeneity between studies, and back-transformed afterwards. Confidence intervals (CIs) were calculated according to the Wilson score method. Heterogeneity was quantified using the I^2^ measure if more than three studies were included. In cases where heterogeneity was encountered, univariate meta-regression analysis was used to explore the influence of alarm symptoms, location of malignancy, and sample size as a source for heterogeneity. Publication bias was assessed using funnel plots, Egger’s test, and Begg’s test. STATA software version 15.1 (StataCorp LLC, College Station, TX, USA) was used for data transformation and meta-analysis. In general, alpha 0.05 defined statistical significance.

We performed subgroup analyses on malignancy detection rate in patients with dyspepsia in absence of alarm symptoms, location of malignancy (gastric *versus* esophageal), and gender (male *versus* female). Prevalence of gastric/esophageal cancer and prevalence in patients with simple dyspepsia and males/females was calculated including OR with 95% CI. In addition, we tested the covariates ‘presence of alarm symptoms’, ‘location of malignancy’, and ‘sample size over 1000’ for a potential source of heterogeneity using univariate meta-regression analysis.

### Quality assessment

We used the modified QUADAS-2 tool to assess the quality of the studies included for primary analysis.^11^ This tool assesses risk of bias on four domains: (a) patient selection (subdomains ‘patient selection’ and ‘appropriateness of population for research question’); (b) reference test (subdomains ‘description of reference test’ and ‘appropriateness of reference test for research question’); (c) index test (subdomains ‘description of index test’ and ‘appropriateness of index test for research question’); (d) patient flow. All domains were scored ‘no risk of bias’, ‘potential risk of bias’, or ‘unclear risk of bias’.

## Results

### Literature search

**Figure 2.**
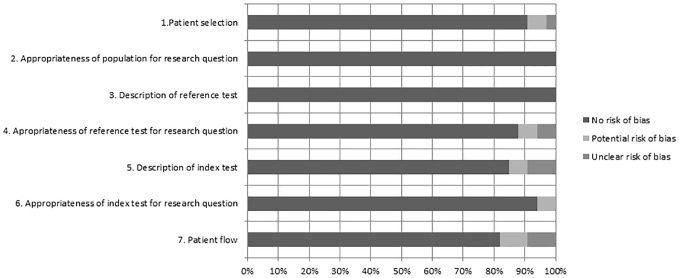
Bar plot of quality assessment according to the modified QUADAS-2, regarding detection of malignancy using upper gastrointestinal endoscopy. Each bar represents a subdomain of one of the four domains: patient selection (1, 2); reference test (3, 4); index test (5, 6); flow and timing (7). The *x*-axis represents all studies included for primary analysis (100%).

The search identified 21,077 studies (Figure 1), of which 33 were included for analysis, representing 11,024 cases of upper GI malignancy (range per study *n* = 6–4362) in at least 346,641 patients in 21 countries and five continents. Characteristics of the included studies for meta-analysis are presented in Table 1. Globally, 24 studies included patients undergoing upper GI endoscopy for all indications,^12–35^ two of which also differentiated between those with and without alarm symptoms.^22,25^ Six studies included patients with dyspepsia only,^36–41^ two included those with dyspepsia or alarm symptoms ^42,43^ and one was limited to patients with alarm symptoms.^44^ Three studies did not report total sample size.^16,26,32^ Overall risk of bias assessed by the QUADAS-2 tool was low. For all domains, there was no risk of bias in >80% of the studies (Figure 2).

**Table 1.** Characteristics of studies included in meta-analysis.

Author	Country	Setting (indication)	Sample size^*^ (% male)	Malignancy type (prevalence %)	Age groups (years)
*North America*					
Canga and Vakil^32^	USA	Secondary (all indications)	Cases with malignancy: 341 (67)	Stomach/esophagus (NA)	<45, 45–55, >55
Lieberman *et al.*^21^	USA	Secondary and tertiary (all indications)	36,357 (45)	Stomach (0.2) Esophagus (0.1)	10 year increment
*South America*					
Uehara *et al.*^43^	Peru	Secondary (dyspepsia or alarm symptoms)	32,388 (NR)	Stomach (1.7)	5 year increment
*Europe*					
Mimica^39^	Bosnia and Herzegovina	Secondary (dyspepsia)	2697 (57)	Stomach (1.3)	5 year increment
Salkic *et al.*^40^	Bosnia and Herzegovina	Tertiary (dyspepsia)	12,884 (55)	Stomach (2.2) Esophagus (0.5)	5 year increment
Dobru *et al.*^16^	Romania	Tertiary (all indications)	Cases with malignancy: 640 (66)	Stomach (NA)	10 year increment
Crouwel *et al.*^33^	The Netherlands	Secondary (all indications)	2006 (46)	Stomach/esophagus (5.2)	5 year increment
Bowrey *et al.*^42^	UK	Tertiary (dyspepsia or dysphagia)	4018 (NR)	Stomach/esophagus (3.1)	10 year increment
Broe *et al.*^30^	Ireland	Secondary (all indications)	4262 (NR)	Stomach (0.6) Esophagus (0.2)	10 year increment
Salo *et al.*^25^	Finland	Tertiary (all indications)	10,061 (37)	Stomach (0.5) Esophagus (0.2)	5 year increment
Sundar *et al.*^27^	UK	Secondary (all indications)	11,145 (NR)	Stomach/esophagus (2.1)	<45, 45–55, >55
Stephens *et al.*^26^	UK	Secondary (all indications)	Cases with malignancy: 300 (NR)	Stomach (NA)	<55, ⩾55
Bolling-Sternevald *et al.*^36^	Sweden	Tertiary (dyspepsia)	799 (48)	Stomach/esophagus (1.2)	⩽40, >40
Qureshi *et al.*^44^	UK	Tertiary (alarm symptoms)	913 (51)	Stomach/esophagus (10.3)	⩽40, 41–50, >50
Boulton-Jones *et al.*^29^	UK	Secondary (all indications)	1000 (NR)	Stomach/esophagus (1.7)	<45, ⩾45
*Asia*					
Liou *et al.*^22^	Taiwan	Tertiary (all indications)	67,662 (NR)	Stomach (0.7)	5 year increment
Wai *et al.*^41^	Singapore	Tertiary (dyspepsia)	5066 (47)	Stomach (0.5)	10 year increment
Li *et al.*^20^	China	Tertiary (all indications)	14,101 (49)	Stomach (1.2) Esophagus (0.3)	<45, 45–60, 60–70, >70
Bai *et al.*^14^	China	Tertiary (all indications)	102,665 (53)	Stomach/esophagus (4.3)	⩽35, 36–54, 55–74, ⩾75
Chan and Goh^34^	Malaysia	Tertiary (all indications)	1076 (45)	Stomach (0.9) Esophagus (0.7)	5 year increment
Mahadeva and Goh^24^	Malaysia	Tertiary (all indications)	1208 (42)	Stomach (0.3) Esophagus (0.3)	<45, ⩾45
Ajlouni^13^	Afghanistan	Secondary (all indications)	289 (59)	Esophagus (22.5)	10 year increment
Fatih *et al.*^37^	Turkey	Tertiary (dyspepsia)	25,037 (NR)	Stomach (51.4) Esophagus (19.7)	10 year increment
Hsu *et al.* ^18^	Taiwan	Secondary (all indications)	2530 (46)	Stomach/esophagus (1.2)	⩽45, >45
Sung *et al.*^28^	Hong Kong	Secondary (all indications)	2627 (NR)	Stomach (0.7) Esophagus (0.1)	<45, ⩾45
*Africa*					
Aduful *et al.*^12^	Ghana	Tertiary (all indications)	6977 (54)	Stomach (2.5)	10 year increment
Gyedu and Yorke^17^	Ghana	Secondary (all indications)	3110 (43)	Stomach (2.0)	20 year increment
Lodenyo *et al.*^23^	Kenya	Secondary (all indications)	768 (63)	Stomach (4.7) Esophagus (9.0)	10 year increment
Diarra *et al.*^15^	Mali	Secondary (all indications)	2250 (NR)	Stomach (4.6)	20 year increment
Bulur *et al.*^31^	Somalia	Secondary (all indications)	306 (68)	Esophagus (18.0)	10 year increment
Kayamba *et al.*^19^	Zambia	Tertiary (all indications)	15,773 (56)	Stomach (2.3) Esophagus (2.7)	<45, 45–60, >60
Gado *et al.*^38^	Egypt	Secondary (dyspepsia)	1400 (51)	Stomach (1.1)	<30, 30–50, >50
Dakubo *et al.*^35^	Ghana	Tertiary (all indications)	1643 (48)	Stomach (3.9)	⩽50, >50

* Sample for which age details are described; may be different from total study sample size.

NA, not applicable; NR, not reported.

### Prevalence of any upper GI malignancy per geographical region

**Figure 3.**
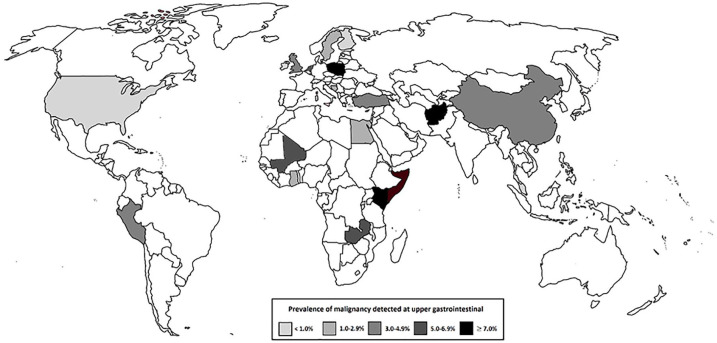
Incidence of upper gastrointestinal malignancy detected in symptomatic patients at upper gastrointestinal endoscopy.

The Asian studies included for analysis documented the overall highest prevalence of upper GI malignancy in a total of 193,467 patients (5.4% 95% CI 2.3–9.7), followed by the studies performed in Africa (*n* = 32,227; 4.4% 95% CI 3.0–6.1), Europe (*n* = 49,785; 2.4% 95% CI 1.4–3.8), South America (*n* = 32,388, 1.7% 95% CI 1.6–1.8), and North America (*n* = 32,045, 0.2% 95% CI 0.2–0.3) (Table 1). Prevalence per country is presented in Figure 3. Within individual continents, there was moderate heterogeneity between studies (I^2^ <65%), which was significant (*p* = 0.04) in Asian studies at age limits of 45 years and 50 years, and in Europe at a 60-year age limit (Supplemental Figure 1). Univariate meta-regression analysis using covariates ‘presence of alarm symptoms’, ‘malignancy location’, and ‘sample size *n* ⩾ 1000’ did not reveal a potential source for heterogeneity (not significant) (Supplemental Table 3). A funnel plot of all included studies showed no clear asymmetry and Egger’s test was nonsignificant (*p* = 0.166). However, as Begg’s test was significant (*p* = 0.006), we identified a potential risk of publication bias (Supplemental Figure 2).

### Malignancy prevalence based on age limits

**Figure 4.**
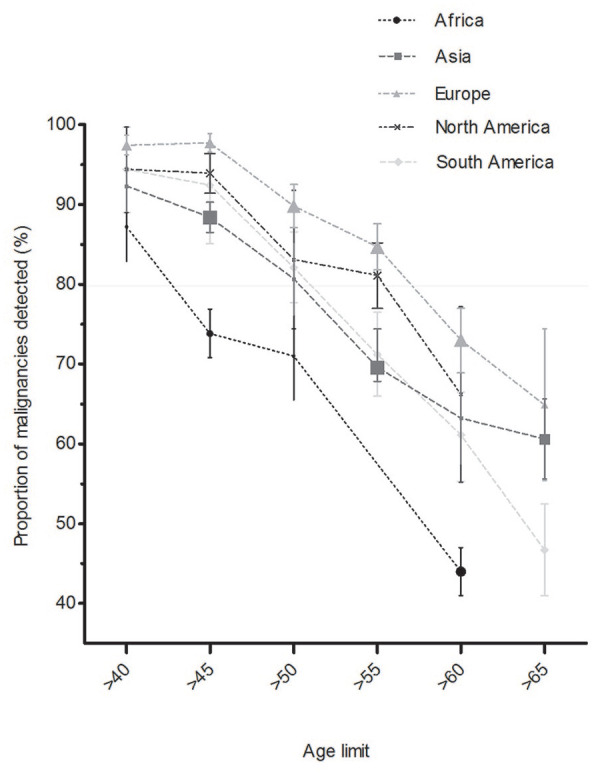
Pooled proportions of malignancies detected above set age limits by continent. Symbol size indicates size of group of patients with malignancy. OR, odds ratio.

Considering the age limits of 40, 45, 50, 55, 60, and 65 years, we found that increasing the age limit at which diagnostic upper GI endoscopy should be performed lowers the detection rate of upper GI malignancy in all continents (Figure 4). This effect was most pronounced in Africa where >0% of malignancies were found only if the age limit was set at ⩾40 years (Supplemental Table 4). In contrast, similar detection rates in South America and Asia were seen at an age limit of ⩾50 years, and in North America and Europe at ⩾55 years.

To detect at least one case of malignancy in North America, 500 endoscopies in patients aged ⩾40 years are needed, against 1884 in patients below 40 years of age (Supplemental Table 6). Considerably fewer endoscopies (*n* = 38) are needed to detect one malignancy in European patients ⩾40 years old, although this was similar for patients aged ⩾45, 50, and 55 years. In patients <40 years of age, over 500 endoscopies are needed in Europe to detect one case of malignancy, this number halved for patients below 55 years of age. The lowest numbers of endoscopies were needed to detect one case of malignancy amongst patients at any age over 40 years in Africa. The number of endoscopies needed in Asia remained over 60 for patients at any age over 40 years, except for those of 55 years of age. These data for 55 years of age were mainly determined by a large Chinese study in an area with high *Helicobacter pylori* prevalence.

**Figure 5.**
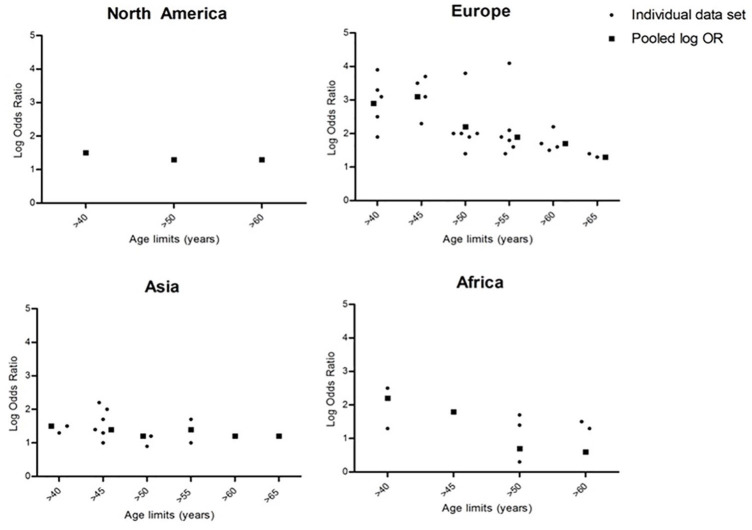
Scatter plot of log OD of the presence of malignancy above and under age for North America, Europe, Asia, and Africa. Small dots represent data of individual studies, squares represent pooled log OR. OR, odds ratio.

In Europe and Africa, we identified a clear decrease in POR with increasing age limits for endoscopy. Figure 5 illustrates this downward trend of the pooled ORs in relation to set age limits in Europe and Africa, whereas a plateau was seen in Asia and North America. Insufficient data were available for South America.

### Malignancy prevalence in patients with dyspepsia

Eight studies separately reported data on patients with dyspepsia without alarm symptoms, that is, one African, three Asian, and four European studies. Independently of location, over 80% of malignancies are detected if endoscopy was reserved for those patients above the age of 55 years. However, studies performed in Asia showed that a cut-off of 50 years is needed to detect near 80% of malignancies (pooled prevalence 95% CI: *n* = 114; 78.1% 69.6–84.7), whereas European studies indicated that this percentage will be detected if an age threshold of 60 years is upheld (pooled prevalence 95% CI: *n* = 34; 88.2% 73.4–95.3). The single available African study provided data for malignancies above and below 50 years of age and showed that only 62.5% (95% CI 38.6–81.5) of malignancies would have been detected if endoscopy was reserved for patients with dyspepsia >50 years of age. Again, POR to detect malignancy decreased with rising age (see Supplemental Table 4).

### Prevalence of gastric cancer and esophageal cancer at specific age limits

Esophageal cancer prevalence was separately reported in 12 studies and gastric cancer in 21. No difference in prevalence by age was seen, as for both cancer types at least 80% of cases were diagnosed at age >40 years in Africa, > 50 years in Asia and North America, and >55 years in Europe (total cases of esophageal cancer: pooled prevalence; 95% CI: *n* = 101; 85.1% 71.3–95.3; *n* = 280; 82.1% 74.1–85.8; *n* = 71; 82.9% 73.2–90.9; *n* = 1082; 88.9% 82.9–93.8; total cases of gastric cancer: pooled prevalence: 95% CI: *n* = 374; 87.8% 83.6–91.4; *n* = 329; 82.2% 77.9–86.2; *n* = 356; 81.6% 77.5–85.3; *n* = 750; 88.9% 79.2–96.0).

### Gender differences in malignancy prevalence

Subgroup analysis by gender revealed that prevalence of malignancy was reported for men and women separately in four studies (Supplemental Table 7). In Africa, malignancies were more frequently discovered in women than in men, mainly because of a higher prevalence of esophageal cancer found in women.^23^ In contrast, prevalence was higher in men compared with women in South America, Europe, and Asia. Age distribution of malignant cases was similar between men and women.

## Discussion

This systematic review and meta-analysis documents the worldwide age distribution at time of upper GI malignancy diagnosis using upper GI endoscopy. We found large intercontinental differences in PORs across various age limits. This global variation has major implications for diagnostic decision making and suggests that guidelines should be tailored to regional epidemiology.

Endoscopic procedures of the upper GI tract are at risk of overuse. Undertaking upper GI endoscopy for an inappropriate indication, according to the American Society for Gastrointestinal Endoscopy (ASGE) and European Panel on the Appropriateness of Gastrointestinal Endoscopy guidelines, is not cost-effective, with an incremental cost-effectiveness of US$301,203.^45^ The low diagnostic yield calls for a strict indication policy. Guidelines defined ‘age’ as an instrument to increase diagnostic yield. Age is a well-known risk factor for both gastric and esophageal cancer.^46^ The prolonged exposure to risk factors (i.e. smoking and alcohol), increased prevalence of obesity in older populations, and lower *H. pylori* infection in younger groups all contribute to lower yield at lower age.^47^ Indeed, the cumulative risk of developing upper GI malignancy at an age between 60 years and 79 years is four times larger compared with patients aged 40–59 years.^48^ Moreover, epidemiologic differences play a key role in how these individual factors contribute to the risk of cancer. For example, *H. pylori* infection prevalence differs widely between continents, with endemic areas like Africa showing an estimated prevalence of 80%, compared with Europe where prevalence of *H. pylori* infection is less than 50%.^49^ Furthermore, in Asian countries, esophageal cancers predominantly include squamous cell carcinomas, of which tobacco and alcohol use are the main risk factors.^50^ In contrast, the majority of patients presenting with esophagus carcinoma in Europe and North America concern adenocarcinoma type and are associated with obesity and reflux disease. The age- and region-related dependency of risk factors is likely to influence malignancy detection rate at different age limits.

Continent-specific age limits should be introduced to improve the yield of upper GI malignancy. In North America, a limit of >55 years of age can be used to detect at least 80% of malignant cases. In contrast, raising the limit to 60 years will lower the yield to 65%. This finding is mirrored by an increased incidence of upper GI malignancy at >55 years of age reported by the USA SEER database.^1,51^ The ASGE guideline recommends an age limits of 50 years. However, this age limit will not result in a higher yield compared with the 55 years of age limit. A lower limit of >50 years of age should be used in South America, to detect at least 80% of cases.

Similar to North America, an age limit of >55 years of age is justified in Europe and aligns with the National Institute for Health and Care Excellence (NICE) guideline recommendations. Limiting upper GI endoscopy to patients >55 years of age will detect 85% of malignancies. In geographical areas with a lower prevalence of upper GI malignancy, a limit of >60 years of age may be justified, particularly in patients who present with dyspepsia in the absence of alarm symptoms, as 88% of malignancies are detected >60 years of age. Global Cancer Observatory (GLOBOCAN) data reveal notable discrepancies in malignancy incidence between North/West Europe and South/East Europe. The latter identifies a gastric cancer incidence of ~25/100.000 at age 50–54 years, compared with 8 in the UK. Despite these low figures, a cost-effectiveness scenario of gastric cancer screening in Eastern and Southern Europe has been suggested.^52^ In our study, which combined both high and low prevalence areas, >70% of cases were among patients ⩾60 years old. It is likely that a majority of <60-year-olds originated from high prevalence areas.

A lower age limit of >50 years should be maintained in Asia, even in those presenting without alarm symptoms. A meta-analysis of six Asian studies confirmed detection of at least 80% of cases in >45-year-olds, but lower rates for patients of 50 years of age. This discrepancy is most likely explained by the larger number of studies we included for analysis. Asian guidelines recommend prevalence-based age limits (40, 45, and 50 years of age in high, intermediate, and low prevalence countries). We found that 250 patients aged below 45 years would have to be scoped to detect one case of malignancy, which indicates potential upper GI endoscopy overuse. An accurate risk assessment contributes to an improved quality of life.^53^

In Africa, symptomatic patients >40 years of age should be investigated to detect at least 80% of malignant cases. In contrast, African guidelines recommend an age limit of 45 years of age. We included only one study providing data of cases above and below 45 years. As detection rate in patients of 45 years or older was similar to that of 50 years or older, we believe that the detection rate at 45 years is an underestimation. This assumption is supported by GLOBOCAN data of 118 Ugandan cases, which revealed a steep increase in incidence of upper GI malignancy after 45 years of age.^1^

Regional common practice should be observed when interpreting these results. Selection of patients for endoscopy is more rigorous in low-prevalence countries, raising the *a priori* chance of detecting upper GI malignancy.^54^ This may explain the similar POR in North America and Asia, and a low number needed to investigate in Europe.

A strength of this meta-analysis was the use of worldwide data, which resulted in a complete overview containing a large number of patients. Moreover, sufficient age limits were used to accommodate all conventional guidelines. Lastly, robust and rigorous statistical analysis was used.

This study comes with several limitations. First, heterogeneity exists between the included studies within continents. Potential causes of heterogeneity could not be identified using subgroup analysis. Variation in endoscopy quality, organization of healthcare, and local risk factors may have caused heterogeneity in our data. Secondly, we identified a risk of publication bias. Also, no relationship between sample size and effect size was seen. Lastly, we used a varying combination of studies to calculate outcomes for each age limit, as many studies omitted data of specific limits. This prevented statistical comparison between age limits within regions.

This systematic review and meta-analysis describes the implications for malignancy detection rate when age limits are used for upper GI endoscopy in a symptomatic population. Intercontinental inequality exists and the use of different age thresholds in local guidelines is crucial. The identified age thresholds should aid in informing future guidelines.

## Supplemental Material

Supplementary_figure_1a – Supplemental material for Systematic review with meta-analysis: age-related malignancy detection rates at upper gastrointestinal endoscopy Supplemental material, Supplementary_figure_1a for Systematic review with meta-analysis: age-related malignancy detection rates at upper gastrointestinal endoscopy by Judith J. de Jong, Marten A. Lantinga, Ina M. E. Thijs, Philip R. de Reuver and Joost P. H. Drenth in Therapeutic Advances in Gastroenterology

Supplementary_figure_1b – Supplemental material for Systematic review with meta-analysis: age-related malignancy detection rates at upper gastrointestinal endoscopy Supplemental material, Supplementary_figure_1b for Systematic review with meta-analysis: age-related malignancy detection rates at upper gastrointestinal endoscopy by Judith J. de Jong, Marten A. Lantinga, Ina M. E. Thijs, Philip R. de Reuver and Joost P. H. Drenth in Therapeutic Advances in Gastroenterology

Supplementary_figure_1c – Supplemental material for Systematic review with meta-analysis: age-related malignancy detection rates at upper gastrointestinal endoscopy Supplemental material, Supplementary_figure_1c for Systematic review with meta-analysis: age-related malignancy detection rates at upper gastrointestinal endoscopy by Judith J. de Jong, Marten A. Lantinga, Ina M. E. Thijs, Philip R. de Reuver and Joost P. H. Drenth in Therapeutic Advances in Gastroenterology

Supplementary_figure_1d – Supplemental material for Systematic review with meta-analysis: age-related malignancy detection rates at upper gastrointestinal endoscopy Supplemental material, Supplementary_figure_1d for Systematic review with meta-analysis: age-related malignancy detection rates at upper gastrointestinal endoscopy by Judith J. de Jong, Marten A. Lantinga, Ina M. E. Thijs, Philip R. de Reuver and Joost P. H. Drenth in Therapeutic Advances in Gastroenterology

Supplementary_figure_1e – Supplemental material for Systematic review with meta-analysis: age-related malignancy detection rates at upper gastrointestinal endoscopy Supplemental material, Supplementary_figure_1e for Systematic review with meta-analysis: age-related malignancy detection rates at upper gastrointestinal endoscopy by Judith J. de Jong, Marten A. Lantinga, Ina M. E. Thijs, Philip R. de Reuver and Joost P. H. Drenth in Therapeutic Advances in Gastroenterology

Supplementary_figure_1f – Supplemental material for Systematic review with meta-analysis: age-related malignancy detection rates at upper gastrointestinal endoscopy Supplemental material, Supplementary_figure_1f for Systematic review with meta-analysis: age-related malignancy detection rates at upper gastrointestinal endoscopy by Judith J. de Jong, Marten A. Lantinga, Ina M. E. Thijs, Philip R. de Reuver and Joost P. H. Drenth in Therapeutic Advances in Gastroenterology

Supplementary_figure_2 – Supplemental material for Systematic review with meta-analysis: age-related malignancy detection rates at upper gastrointestinal endoscopy Supplemental material, Supplementary_figure_2 for Systematic review with meta-analysis: age-related malignancy detection rates at upper gastrointestinal endoscopy by Judith J. de Jong, Marten A. Lantinga, Ina M. E. Thijs, Philip R. de Reuver and Joost P. H. Drenth in Therapeutic Advances in Gastroenterology

Supplementary_files – Supplemental material for Systematic review with meta-analysis: age-related malignancy detection rates at upper gastrointestinal endoscopy Supplemental material, Supplementary_files for Systematic review with meta-analysis: age-related malignancy detection rates at upper gastrointestinal endoscopy by Judith J. de Jong, Marten A. Lantinga, Ina M. E. Thijs, Philip R. de Reuver and Joost P. H. Drenth in Therapeutic Advances in Gastroenterology
