# Supplementary figures and images for: Systematic review with meta-analysis: age-related malignancy detection rates at upper gastrointestinal endoscopy
Source: Ther Adv Gastroenterol. 2020 Nov 4;13:1756284820959225. doi: 10.1177/1756284820959225 (PMC7645776; doi:10.1177/1756284820959225)

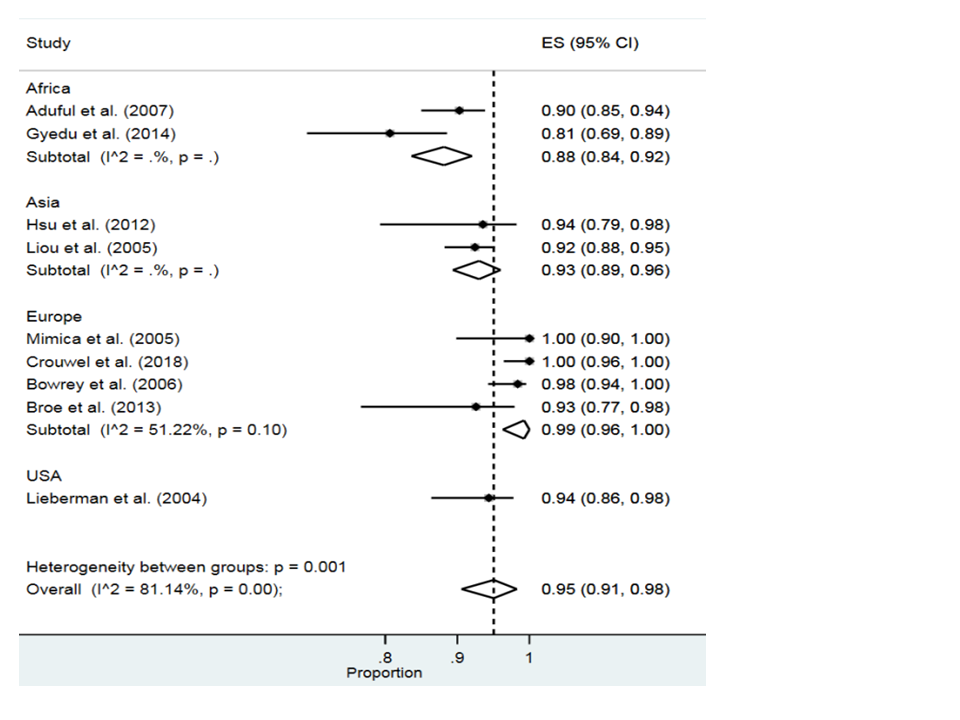

Supplement: Supplementary_figure_1a – Supplemental material for Systematic review with meta-analysis: age-related malignancy detection rates at upper gastrointestinal endoscopy [file Supplementary_figure_1a.TIF]

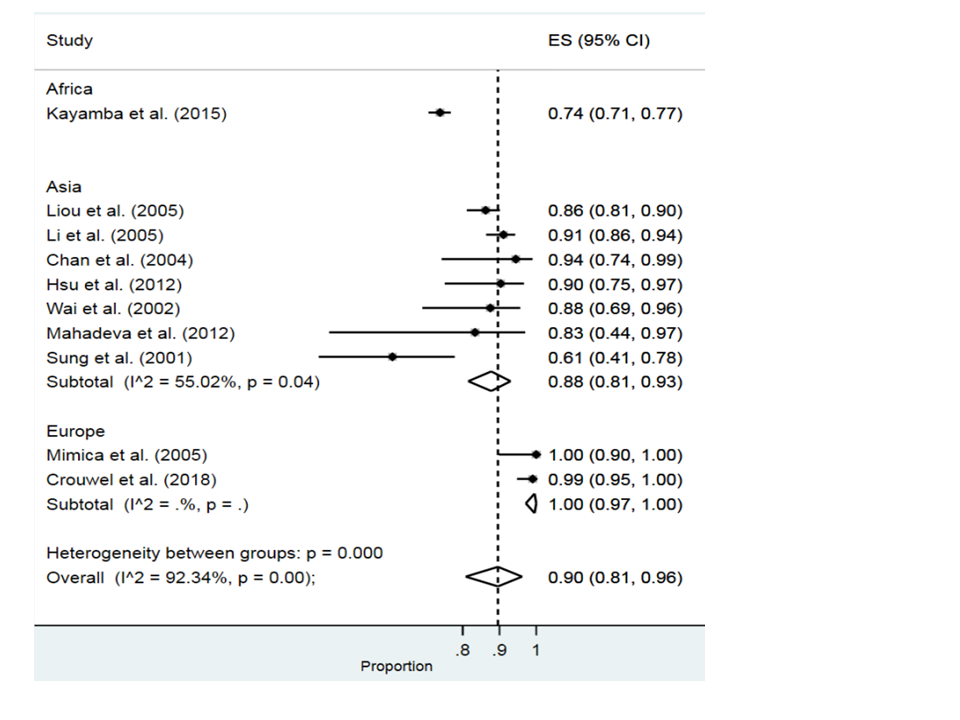

Supplement: Supplementary_figure_1b – Supplemental material for Systematic review with meta-analysis: age-related malignancy detection rates at upper gastrointestinal endoscopy [file Supplementary_figure_1b.TIF]

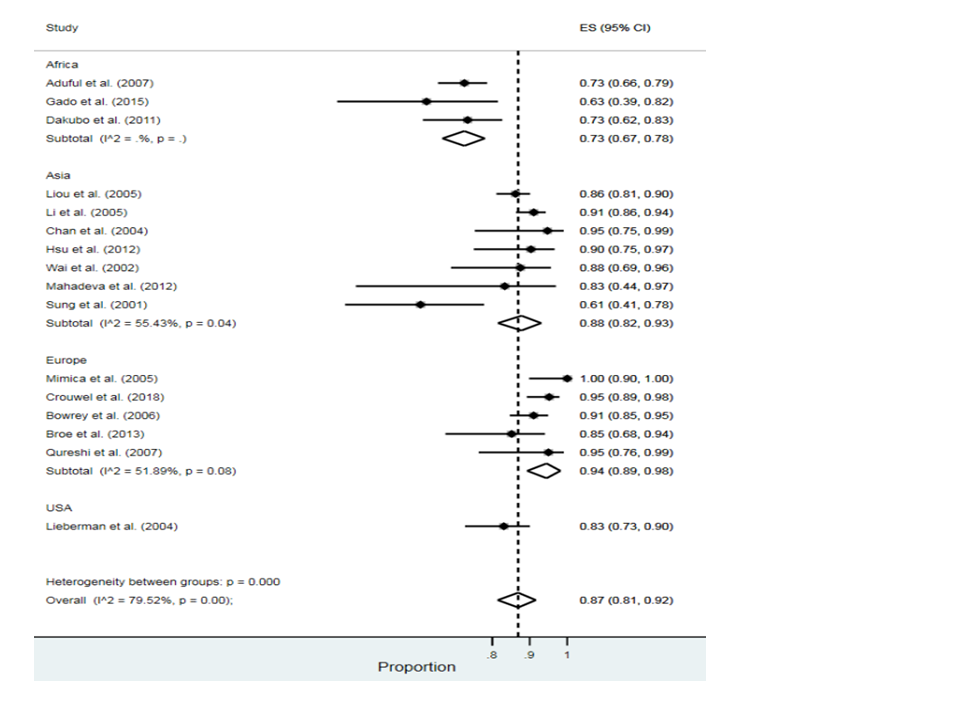

Supplement: Supplementary_figure_1c – Supplemental material for Systematic review with meta-analysis: age-related malignancy detection rates at upper gastrointestinal endoscopy [file Supplementary_figure_1c.TIF]

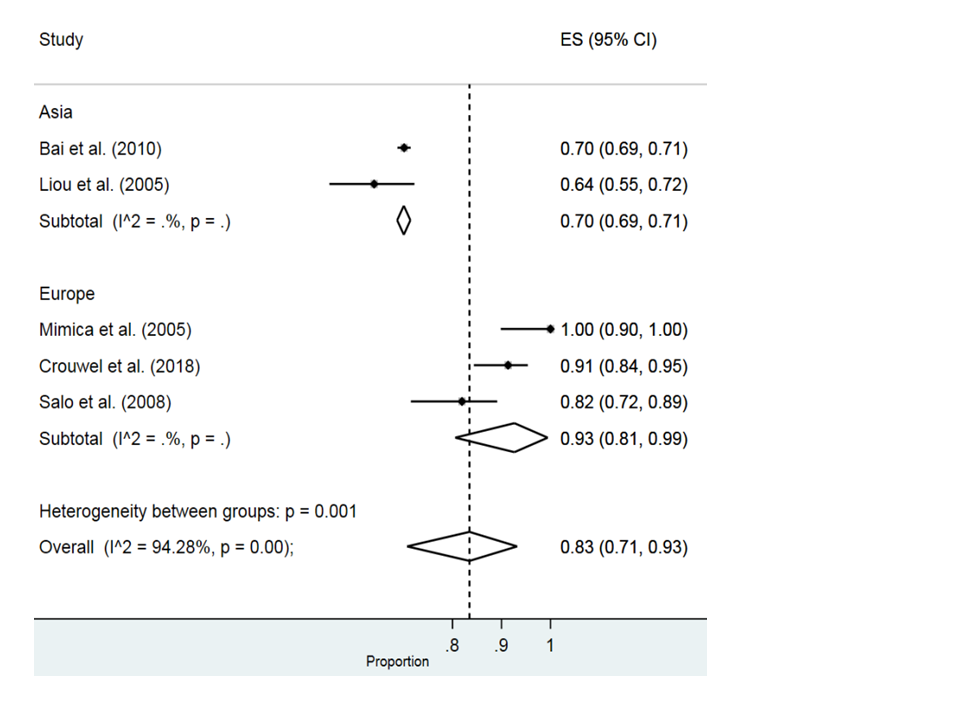

Supplement: Supplementary_figure_1d – Supplemental material for Systematic review with meta-analysis: age-related malignancy detection rates at upper gastrointestinal endoscopy [file Supplementary_figure_1d.TIF]

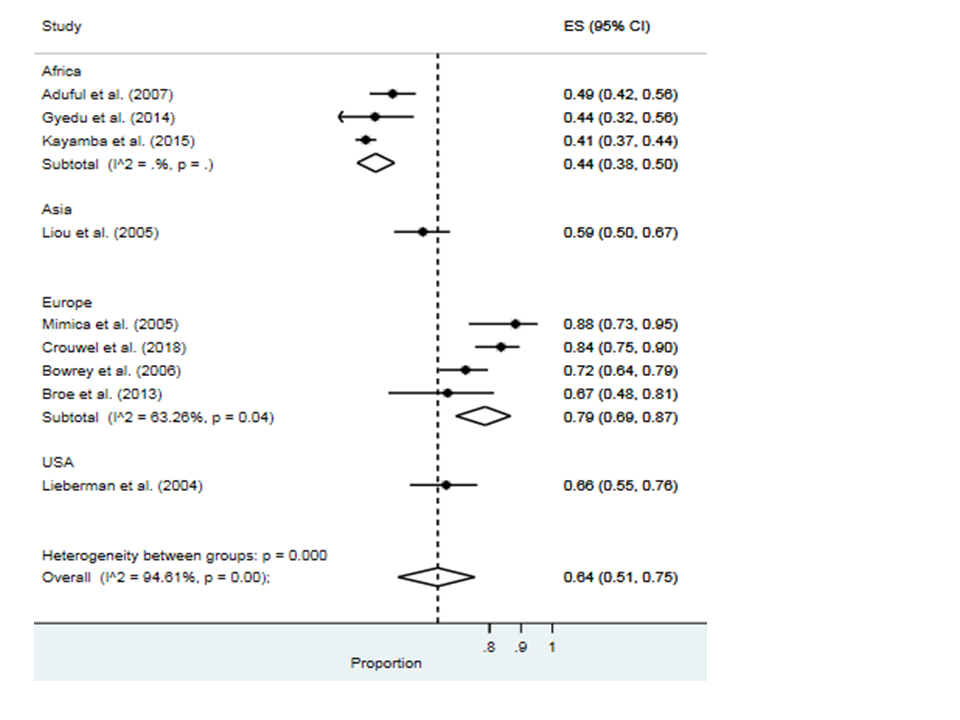

Supplement: Supplementary_figure_1e – Supplemental material for Systematic review with meta-analysis: age-related malignancy detection rates at upper gastrointestinal endoscopy [file Supplementary_figure_1e.TIF]

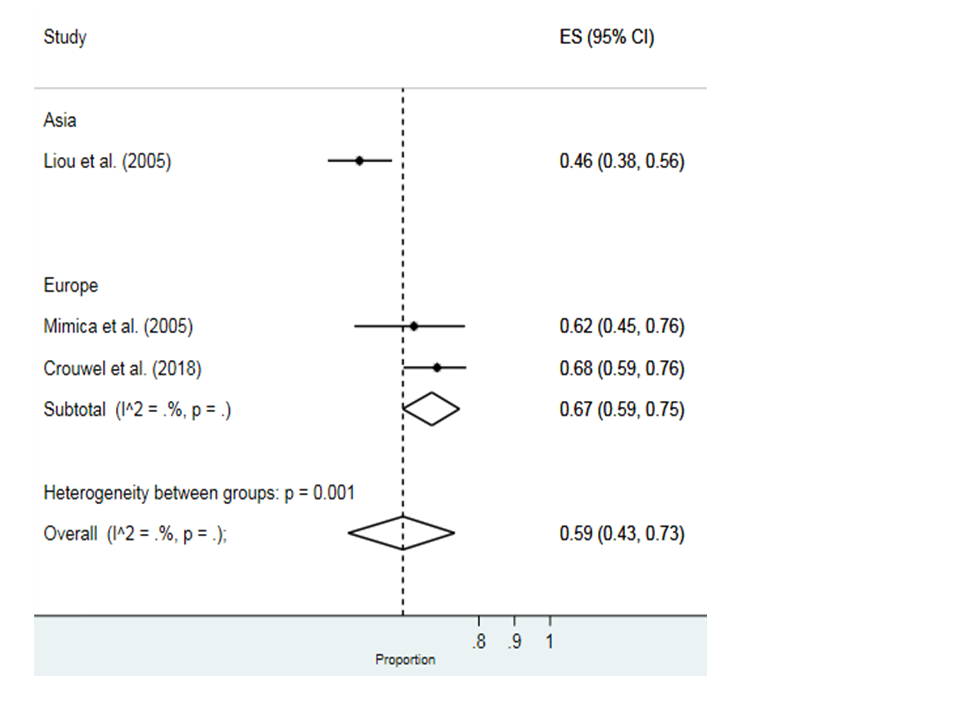

Supplement: Supplementary_figure_1f – Supplemental material for Systematic review with meta-analysis: age-related malignancy detection rates at upper gastrointestinal endoscopy [file Supplementary_figure_1f.TIF]

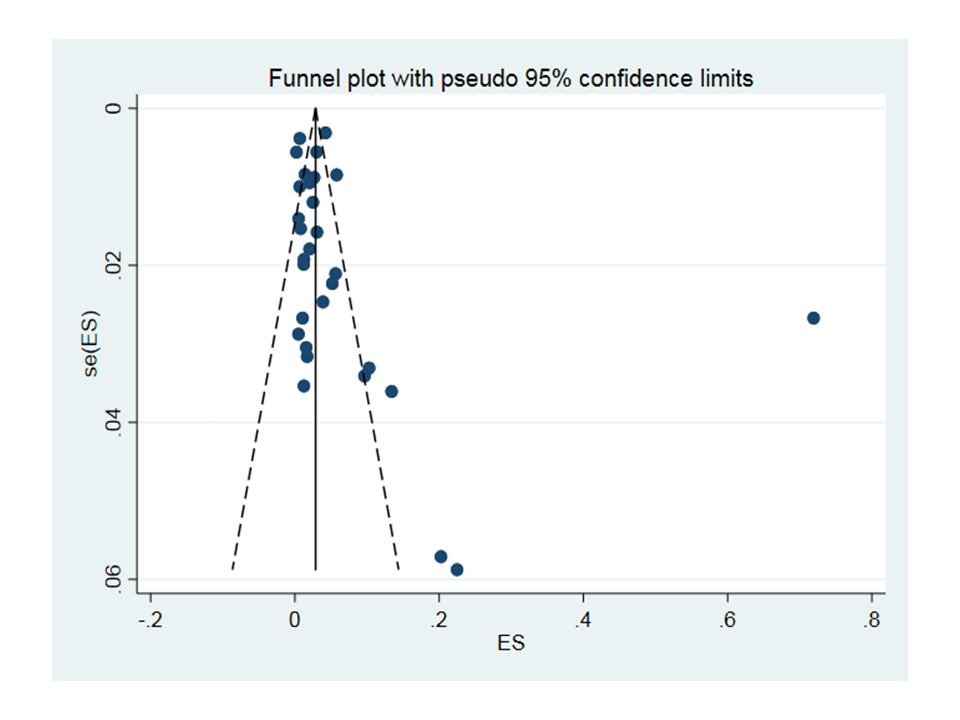

Supplement: Supplementary_figure_2 – Supplemental material for Systematic review with meta-analysis: age-related malignancy detection rates at upper gastrointestinal endoscopy [file Supplementary_figure_2.tif]
