# Supplementary material for: Systematic review with meta-analysis: age-related malignancy detection rates at upper gastrointestinal endoscopy
Source: Ther Adv Gastroenterol. 2020 Nov 4;13:1756284820959225. doi: 10.1177/1756284820959225 (PMC7645776; doi:10.1177/1756284820959225)
Supplement: Supplementary_files – Supplemental material for Systematic review with meta-analysis: age-related malignancy detection rates at upper gastrointestinal endoscopy [file Supplementary_files.pdf]

Supplementary table 1.

Section/topic	#	Checklist item	Reported on page #
**TITLE**			
Title	1	Identify the report as a systematic review, meta-analysis, or both.	1
**ABSTRACT**			
Structured summary	2	Provide a structured summary including, as applicable: background; objectives; data sources; study eligibility criteria, participants, and interventions; study appraisal and synthesis methods; results; limitations; conclusions and implications of key findings; systematic review registration number.	2
**INTRODUCTION**			
Rationale	3	Describe the rationale for the review in the context of what is already known.	3
Objectives	4	Provide an explicit statement of questions being addressed with reference to participants, interventions, comparisons, outcomes, and study design (PICOS).	3
**METHODS**			
Protocol and registration	5	Indicate if a review protocol exists, if and where it can be accessed (e.g., Web address), and, if available, provide registration information including registration number.	4
Eligibility criteria	6	Specify study characteristics (e.g., PICOS, length of follow-up) and report characteristics (e.g., years considered, language, publication status) used as criteria for eligibility, giving rationale.	4-5
Information sources	7	Describe all information sources (e.g., databases with dates of coverage, contact with study authors to identify additional studies) in the search and date last searched.	4
Search	8	Present full electronic search strategy for at least one database, including any limits used, such that it could be repeated.	Suppl. Table S2
Study selection	9	State the process for selecting studies (i.e., screening, eligibility, included in systematic review, and, if applicable, included in the meta-analysis).	4
Data collection process	10	Describe method of data extraction from reports (e.g., piloted forms, independently, in duplicate) and any processes for obtaining and confirming data from investigators.	5

Data items	11	List and define all variables for which data were sought (e.g., PICOS, funding sources) and any assumptions and simplifications made.	5
Risk of bias in individual studies	12	Describe methods used for assessing risk of bias of individual studies (including specification of whether this was done at the study or outcome level), and how this information is to be used in any data synthesis.	6
Summary measures	13	State the principal summary measures (e.g., risk ratio, difference in means).	5-6
Synthesis of results	14	Describe the methods of handling data and combining results of studies, if done, including measures of consistency (e.g., I^2) for each meta-analysis.	5-6
Risk of bias across studies	15	Specify any assessment of risk of bias that may affect the cumulative evidence (e.g., publication bias, selective reporting within studies).	5-6
Additional analyses	16	Describe methods of additional analyses (e.g., sensitivity or subgroup analyses, meta-regression), if done, indicating which were pre-specified.	6
**RESULTS**			
Study selection	17	Give numbers of studies screened, assessed for eligibility, and included in the review, with reasons for exclusions at each stage, ideally with a flow diagram.	7
Study characteristics	18	For each study, present characteristics for which data were extracted (e.g., study size, PICOS, follow-up period) and provide the citations.	7
Risk of bias within studies	19	Present data on risk of bias of each study and, if available, any outcome level assessment (see item 12).	7
Results of individual studies	20	For all outcomes considered (benefits or harms), present, for each study: (a) simple summary data for each intervention group (b) effect estimates and confidence intervals, ideally with a forest plot.	7 and suppl figure S1
Synthesis of results	21	Present results of each meta-analysis done, including confidence intervals and measures of consistency.	7-9
Risk of bias across studies	22	Present results of any assessment of risk of bias across studies (see Item 15).	7
Additional analysis	23	Give results of additional analyses, if done (e.g., sensitivity or subgroup analyses, meta-regression [see Item 16]).	8-9
**DISCUSSION**			
Summary of evidence	24	Summarize the main findings including the strength of evidence for each main outcome; consider their relevance to key groups (e.g., healthcare providers, users, and policy makers).	9-10

Limitations	25	Discuss limitations at study and outcome level (e.g., risk of bias), and at review-level (e.g., incomplete retrieval of identified research, reporting bias).	11-12
Conclusions	26	Provide a general interpretation of the results in the context of other evidence, and implications for future research.	12
**FUNDING**			
Funding	27	Describe sources of funding for the systematic review and other support (e.g., supply of data); role of funders for the systematic review.	12

Supplemenatry table 2.

	Upper GI malignancy	***Upper GI endoscopy***	***Upper GI symptoms***
MEDLINE	("Gastrointestinal Neoplasms"[Mesh:noexp] OR "Esophageal Neoplasms"[Mesh] OR "Stomach Neoplasms"[Mesh] OR ((Cancer[ALL] OR cancers[ALL] OR Malignant[ALL] OR Malignancy[ALL] OR malignancies[ALL] OR Neoplasm[ALL] OR neoplasms[ALL] OR Carcinoma[ALL] OR carcinomas[ALL] OR Adenoma[ALL] OR adenomas[ALL] OR Tumor[ALL] OR Tumors[ALL] OR Tumour[ALL] OR Tumours[ALL]) AND (stomach[ALL] OR gastric[ALL] OR “upper gastrointestinal”[ALL] OR esophagus[ALL] OR esophageal[ALL] OR oesophagus[ALL] OR oesophageal[ALL] OR “upper GI”[ALL] OR junction[ALL])))	("Endoscopy, Digestive System"[Mesh:noexp] OR "Endoscopy, Gastrointestinal"[Mesh:noexp] OR "Duodenoscopy"[Mesh] OR "Esophagoscopy"[Mesh] OR "Gastroscopy"[Mesh] OR “gastrointestinal endoscopy”[tiab] OR “GI endoscopy”[tiab] OR gastroscopy[tiab] OR OGD[tiab] OR EGD[tiab] OR oesophagogastroduodenoscopy[tiab] OR esophagogastroduodenoscopy[tiab] OR gastroduodenoscopy[tiab] OR “upper endoscopy”[tiab] OR ((endoscopy[tiab] OR endoscopic[tiab]) AND (oesophagus[tiab] OR oesophageal[tiab]OR esophagus[tiab] OR esophageal[tiab] OR gastric[tiab] OR stomach[tiab] OR duodenal[tiab] OR gastrointestinal[tiab])))	("Dyspepsia"[MeSH] OR "upper gastrointestinal tract"[MeSH] OR "stomach"[MeSH] OR "Gastroesophageal Reflux"[MeSH] OR "Stomach Diseases"[MeSH:noexp] OR "Heartburn"[MeSH] OR Indigestion[tiab] OR Dyspepsia[tiab] OR Dyspeptic[tiab]OR “gastrointestinal symptom”[tiab] OR “gastrointestinal symptoms”[tiab]OR “GI symptom”[tiab] OR “GI symptoms”[tiab] OR “Stomach complaint”[tiab] OR “Stomach complaints”[tiab]OR “gastric pain”[tiab] OR “epigastric pain”[tiab] OR reflux[tiab] OR GORD[tiab] OR GERD[tiab] OR Heartburn[tiab] OR Pyrosis[tiab] OR “epigastric pain”[tiab] OR “non ulcer dyspepsia”[tiab] OR “non-ulcer dyspepsia”[tiab] OR “Functional Gastrointestinal Disorder”[tiab] OR “Functional Gastrointestinal Disorders”[tiab] OR “Functional Dyspepsia”[tiab] OR “alarm symptom”[tiab] OR “alarm symptoms”[tiab] OR “alarming symptom”[tiab] OR “alarming symptoms”[tiab] OR “sinister symptom”[tiab] OR “sinister symptoms”[tiab] OR “alarm feature”[tiab] OR “alarm features”[tiab] OR “alarming

			feature”[tiab] OR “alarming features”[tiab] OR “red flags”[tiab] OR “red flag”[tiab] OR “diagnostic yield”[tiab])
EMBASE	Exp digestive system cancer/ OR exp digestive system tumor/ OR exp esophagus cancer/ OR exp stomach cancer/ OR exp gastrointestinal tumor/ OR exp gastrointestinal stromal tumor/ OR ((Gastro?intestinal ADJ3 neoplasm*) OR (Gastro?intestinal ADJ3 tumo?r*) OR (Gastro?intestinal ADJ3 malignan*) OR (Gastro?intestinal ADJ3 cancer*) OR (Gastro?intestinal ADJ3 Carcinoma*) OR (Gastro?intestinal ADJ3 Adenoma*) OR (?esophag* ADJ3 neoplasm*) OR (?esophag* ADJ3 tumo?r*) OR (?esophag* ADJ3 malignan*) OR (?esophag* ADJ3 cancer*) OR (?esophag* ADJ3 Carcinoma*) OR (?esophag* ADJ3 Adenoma*) OR (stomach ADJ3 neoplasm*) OR (stomach ADJ3 tumo?r*) OR (stomach ADJ3 malignan*) OR (stomach ADJ3 cancer*) OR (stomach ADJ3 Carcinoma*) OR (stomach ADJ3 Adenoma*) OR (gastric ADJ3 neoplasm*) OR (gastric ADJ3 tumo?r*) OR (gastric ADJ3 malignan*) OR (gastric ADJ3 cancer*) OR (gastric ADJ3 Carcinoma*) OR (gastric ADJ3 Adenoma*) OR (upper gastro?intestinal ADJ3 neoplasm*) OR (upper gastro?intestinal ADJ3 tumo?r*) OR (upper gastro?intestinal ADJ3 malignan*) OR (upper gastro?intestinal ADJ3 cancer*) OR (upper gastro?intestinal ADJ3 Carcinoma*) OR (upper gastro?intestinal ADJ3 Adenoma*) OR (upper GI ADJ3 neoplasm*) OR (upper GI ADJ3 tumo?r*) OR (upper GI ADJ3 malignan*) OR (upper GI ADJ3 cancer*) OR (upper GI ADJ3 Carcinoma*) OR (upper GI ADJ3 Adenoma*).ti,ab,kw.)	Exp gastroscopie/ or exp gastrointestinal endoscopy/ or exp esophagoscopy or digestive tract endoscopy/ or endoscopy/ or esophagogastroduodenoscopy/ or exp duodenoscopy/ or (Gastroscop* or (gastro?intestinal ADJ3 endoscop*) or ?esophagoscop* or (digestive tract ADJ3 endoscop*) or endoscop* or ? esophagogastroduodenoscop* or duodenoscop* or (upper GI ADJ3 endoscop*) or (gastric* ADJ3 endoscop*) or (endoscop* ADJ4 stomach) or (endoscop* ADJ4 ?esophag*) or (endoscop* ADJ4 duoden*)).ti,ab,kw.	Exp dyspepsia/ OR stomach disease/ or digestive system disease/ or stomach discomfort/ or stomach function disorder/ or stomach irritation/ or stomach pain/ or stomach spasm/ or exp heartburn/ or indigestion/ or exp epigastric pain/ or digestive system function disorder/ or digestive system disease/ or bloating/ or esophagus function disorder/ or gastrointestinal motility disorder/ or gastrointestinal reflux/ or intestine function disorder/ or stomach function disorder/ or (Dyspep* or (stomach ADJ3 disease*) or (digestive ADJ3 system ADJ3 disease*) or (stomach ADJ3 discomfort) or (stomach ADJ3 function* ADJ3 disorder*) or (stomach ADJ3 irritation*) or (stomach ADJ3 pain*) or (stomach ADJ3 complaint*) or (heartburn) or (indigest*) or (epigastric ADJ3 pain) or bloating or (gastro?intestinal ADJ3 motility ADJ3 disorder*) or (stomach ADJ3 disorder*) or (gastric ADJ3 pain) or (gastric ADJ3 complaint*) or functional gastro?intestinal or functional dyspepsia or upper gastro?intestinal symptom*).ti,ab,kw.

Web of science	TS = (Gastrointestinal Neoplasms OR Esophageal Neoplasms OR Stomach Neoplasms OR ((Cancer OR cancers OR Malignant OR Malignancy OR malignancies OR Neoplasm OR neoplasms OR Carcinoma OR carcinomas OR Adenoma OR adenomas OR Tumor OR Tumors OR Tumour OR Tumours) AND (stomach OR gastric OR upper gastrointestinal OR esophagus OR esophageal OR oesophagus OR oesophageal OR upper GI OR junction)))	TS = (Duodenoscopy OR Esophagoscopy OR Gastroscopy OR gastrointestinal endoscopy OR GI endoscopy OR gastroscopy OR OGD OR EGD OR oesophagogastroduodenoscopy OR esophagogastroduodenoscopy OR gastroduodenoscopy OR upper endoscopy OR ((endoscopy OR endoscopic) AND (oesophagus OR oesophagealOR esophagus OR esophageal OR gastric OR stomach OR duodenal OR gastrointestinal)))	TS = (Dyspepsia OR upper gastrointestinal tract OR stomach OR Gastroesophageal Reflux OR Heartburn OR Indigestion OR Dyspepsia OR Dyspeptic OR gastrointestinal symptom OR gastrointestinal symptoms OR GI symptom OR GI symptoms OR Stomach complaint OR Stomach complaints OR gastric pain OR epigastric pain OR reflux OR GORD OR GERD OR Heartburn OR Pyrosis OR epigastric pain OR non ulcer dyspepsia OR non-ulcer dyspepsia OR Functional Gastrointestinal Disorder OR Functional Gastrointestinal Disorders OR Functional Dyspepsia OR alarm symptom OR alarm symptoms OR alarming symptom OR alarming symptoms OR sinister symptom OR sinister symptoms OR alarm feature OR alarm features OR alarming feature OR alarming features OR red flags OR red flag OR diagnostic yield)

Supplementary table 3.

	**REGION**		**SYMPTOMS**		**CANCER LOCATION**		**SAMPLE SIZE >1,000**	
**AGE LIMIT**	*OR (SE)*	*p*	*OR (SE)*	*p*	*OR (SE)*	*p*	*OR (SE)*	*p*
**>40 YR**	0.88 (0.19)	0.57	2.40 (1.82)	0.29	0.83 (0.29)	0.60	1.93 (1.71)	0.48
**>45 YR**	0.77 (0.24)	0.43	2.15 (1.63)	0.34	0.86 (0.40)	0.75	NA	NA
**>50 YR**	1.03 (0.22)	0.90	1.48 (0.58)	0.34	0.72 (0.16)	0.18	0.99 (1.18)	0.99
**>55 YR**	0.65 (0.38)	0.51	13.86 (20.39)	0.17	0.73 (0.09)	0.08	NA	NA
**>60 YR**	1.41 (0.57)	0.42	2.37 (2.23)	0.39	0.62 (0.30)	0.35	NA	NA
**>65 YR**	0.85 (0.23)	0.66	1.03 (0.40)	0.96	0.93 (0.13)	0.67	NA	NA

Supplementary table 4.

	Age cutoff (years)					
	**40**	**45**	**50**	**55**	**60**	**65**
**North America**						
% of cases above age limit (95%CI)	94.4 (89.0-99.7)	93.9 (91.4-96.4)	83.1 (74.4-91.8)	81.1 (77.0-85.2)	66.2 (55.2-77.2)	NA
Number of malignancies	*71*	*344*	*71*	*344*	*71*	
Prevalence Odds Ratio (95% CI)	*4.4 (0.5-12.0)*	*NA*	*3.6 (1.9-6.7)*	*NA*	*3.8 (2.4-6.3)*	*NA*
Number of malignancies	*71*		*71*		*71*	
To detect 1 case > / < age limit	*500 / 1,884*	*NA*	*333 / 1,280*	*NA*	*250 / 1,003*	*NA*
**South America**						
% of cases above age limit (95%CI)	*94.4 (89.0-99.7)*	*88.8 (85.1-92.4)*	*82.1 (77.7-86.6)*	*71.2 (66.0-76.5)*	*61.1 (55.3-66.5)*	*46.7 (41.0-52.5)*
Number of malignancies	*285*	*285*	*285*	*285*	*285*	*285*
Prevalence Odds Ratio (95% CI)	NA	NA	NA	NA	NA	NA
Number of malignancies						
To detect 1 case > / < age limit	NA	NA	NA	NA	NA	NA

**Europe**						
% of cases above age limit (95%CI)	97.4 (96.2-98.7)	97.7 (96.6-98.9)	89.8 (87.1-92.5)	84.7 (81.8-87.6)	73.0 (68.9-77.0)	64.9 (55.4-74.4)
Number of malignancies	*1,383*	*799*	*1,445*	*1,082*	*1,351*	*554*
Prevalence Odds Ratio (95% CI)	12.9 (5.5-30.2)	15.3 (7.0-33.4)	7.2 (5.0-10.6)	5.7 (4.4-7.5)	5.1 (3.8-6.7)	3.8 (2.6-5.5)
Number of malignancies	*370*	*343*	*390*	*449*	*288*	*138*
Prevalence Odds Ratio (95% CI)	21.4 (1.3-352.9)	37.6 (7.5-187.2)	8.6 (4.3-17.4)	6.5 (4.4-9.8)	9.4 (3.3-26.6)	3.6 (1.8-7.3)
(dyspepsia only)						
Number of malignancies	*116*	*116*	*116*	*155*	*34*	*34*
To detect 1 case > / < age limit	38 / 501	29 / 715	32 / 252	32 / 218	25 / 140	19 / 81
**Asia**						
% of cases above age limit (95%CI)	92.3 (89.1-95.9)	88.4 (86.5-90.3)	80.7 (76.2-85.1)	69.6 (67.8-74.4)	*63.2 (57.5-68.8)*	*60.6 (55.6-65.6)*
Number of malignancies	*314*	*5,710*	*394*	*5,525*	*394*	*1,163*
Prevalence Odds Ratio (95% CI)	4.6 (3.5-4.7)	4.0 (3.0-5.4)	3.2 (2.2-4.8)	5.5 (5.2-5.8)	*3.3 (2.3-4.8)*	*3.2 (2.2-4.7)*
Number of malignancies	*256*	*529*	*145*	*4,476*	*114*	*114*
Prevalence Odds Ratio (95% CI)		5.5 (3.2-9.5)	3.5 (2.2-5.5)	2.7 (1.8-4.0)	3.3 (2.3-4.8)	3.2 (2.2-4.6)
(dyspepsia only)						

Number of malignancies		*138*	*114*	*114*	*114*	*114*
To detect 1 case > / < age limit	67 / 288	67 / 252	91 / 252	14 / 102	*83 / 266*	*71 / 231*
**Africa**						
% of cases above age limit (95%CI)	87.2 (82.9-91.6)	*73.8 (70.8-76.9)*	71.0 (65.5-76.4)	NA	44.0 (41.0-47.0)	NA
Number of malignancies	*442*	*806*	*357*		*1,248*	
Prevalence Odds Ratio (95% CI)	7.7 (5.2-11.4)	*5.9 (5.0-6.9)*	2.0 (1.5-2.7)	NA	2.3 (2.0-2.7)	NA
Number of malignancies	*237*	*806*	*255*		*1,043*	
To detect 1 case > / < age limit	22 / 163	8 / 43	22 / 68	NA	15 / 27	NA

Malignancy detection for each age limit according to continent. CI: Confidence interval; NA: Not applicable due to insufficient data available. Italic values are based on a single study.

Supplementary table 5.

	**Men**	**Women**
**North America**	NA	NA
OR (95% CI)	*NA*	
**South America**	4.7%	2.3%

OR (95% CI)	*2.1 (1.8-2.3); p < 0.001*	
**Europe**	1.8%	0.5%
OR (95% CI)	*3.6 (1.5-8.7); p < 0.001*	
**Asia**	57.6%*	42.4%*
OR (95% CI)	*NA*	
**Africa**	11.2%	17.5%
OR (95% CI)	*0.6 (0.4-0.9); p < 0.001*	

Figure legends

Supplementary figure 1. Proportion of malignancies found over age 40 (a), 45 (b), 50 (c), 55 (d), 60 (e), and 65 (f) by upper GI endoscopy, stratified by region.

Supplementary figure 2. Funnel plot of included study to detect potential publication bias.
